# Epigenetic Clocks Can Predict Age in the Largemouth Bass‐Complex Despite Spatial and Sexual Variation

**DOI:** 10.1111/eva.70328

**Published:** 2026-10-14

**Authors:** Steven T. Gardner, Daniel J. Daugherty, Dijar Lutz‐Carrillo, Brenda Pracheil, Kristine N. Moody

**Affiliations:** ^1^ Environmental Sciences Division Oak Ridge National Laboratory Oak Ridge Tennessee USA; ^2^ Heart of the Hills Fisheries Science Center Texas Parks and Wildlife Department Mountain Home Texas USA; ^3^ Analytical Services Laboratory Texas Parks and Wildlife Department San Marcos Texas USA; ^4^ Earth Systems Science Division Pacific Northwest National Laboratory Richland Washington USA

**Keywords:** biological age, DNA methylation, environmental variation, epigenetic age, largemouth bass

## Abstract

Sustainable fisheries rely on balancing harvests with ecological impacts. Despite the importance of species' demographic profiles, many assessments forgo age due to methodological complexities. Developing non‐lethal methods for age determination can improve stock assessments to ensure future sustainability. Epigenetic signals, specifically DNA methylation, are dynamic and considered to be associated with decreasing genomic stability with age. Subsequently, the modelling of methylation percentages at CpG loci has been utilized to accurately predict chronological age in many organisms, including several fish species. We examined the influence of age (1–14 years), location (Texas versus Tennessee), and sex on DNA methylation patterns of members of the largemouth bass‐complex (LBc), consisting of largemouth bass (*Micropterus nigricans*), Florida bass ( *Micropterus salmoides* ), and their resulting hybrids, all recreational and commercially important species. As DNA methylation patterns are influenced by sex and environmental signals, we accounted for this when evaluating aging patterns in the LBc. Our results indicate global methylation patterns decrease with age, indicating potential genomic instability, and vary with sex and location. Non‐random changes also occur involving physiological pathways of diverse function. Accounting for this variability, aging clocks using single or regions of CpG loci were derived from 124 LBc samples and validated using sets of naïve (*n* = 32) individuals, providing improved estimates over other non‐lethal aging methods. Our results reveal insights into dynamic methylome patterns in the LBc, and when accounting for variability, informative and accurate age estimations can be determined with non‐lethal molecular methods.

## 1 Introduction

A common goal of general fisheries management practices is the improvement of recreational fishing (Scheufele and Pascoe 2022). Recreational fishing constitutes a large commercial industry (Ditton et al. 2002; Brownscombe et al. 2019), but care must be given to avoid exploitation of fish populations and negative impacts to aquatic environments. For example, unregulated fishing may remove specific age‐classes and negatively impact population resilience (Griffiths et al. 2024). To sustain recreational and commercial fishing practices with increased interest and demand, balancing harvests with ecological impacts is critical (Porath et al. 2021). An additional goal of many recreational fisheries is the generation of fast growing “trophy sized” individuals (Chen et al. 2003), with both aims being heavily reliant on informed population age demography estimations (Dotson et al. 2013). Such efforts have been given towards many freshwater species (Arterburn et al. 2002; Bain 1987; Fabrizio et al. 2021; Reed and Parsons 1999; Withers et al. 2017), including largemouth bass (Gwinn and Allen 2010; Kerns et al. 2015; Philipp et al. 1997), which have both ecological and economic importance (Chen et al. 2024).

The largemouth bass (*Micropterus nigricans*) is a highly valued native predatory sport fish associated with a large commercial industry (Snellings 2015). This species has a natural range extending from the St. Lawrence River in the northeastern United States to northern Mexico (Brown et al. 2009) and a worldwide distribution resulting from introductions for recreational fisheries and sustenance (Bae et al. 2018; Long and Seguy 2024). Efforts to increase growth and size distributions of largemouth bass for fisheries and angling purposes have involved stocking Florida bass ( *Micropterus salmoides* ) in many regions beyond their native range of peninsular Florida (MacEina and Murphy 1992), leading to the introgression of Florida genes into many populations (Dunham et al. 1992; Gelwick et al. 1995; Hargrove et al. 2019). This has resulted in many fisheries being dominated by hybrid individuals belonging to the largemouth bass complex (hereafter referred to as LBc) (Lutz‐Carrillo et al. 2023). LBc fry experience rapid growth, which has been positively correlated with air and water temperatures (Scott 1973). Rates of further growth and resulting size‐to‐age relationships vary based on environmental conditions (Coutant and Deangelis 1983; Meador and Kelso 1990; White and Wahl 2020). Additionally, females exhibit slower growth but may reach larger adult sizes compared to males (Beamish et al. 2005). The average lifespan for LBc members can exceed 15 years (Beamesderfer and North 1995), although this too varies with habitat and environment (Finigan and Marschall 2025; Neal 2003).

A prerequisite of sustainable fisheries practices may involve the protection of older age classes, as offspring from these individuals may possess greater fitness compared to those from younger cohorts (Berkeley et al. 2004; Hixon et al. 2014). Such strategies may involve the implementation of strict slot limits, size restriction limits, and enforcing catch‐and‐release practices to minimize stress, injury, and mortality (Hixon et al. 2014; Cooke and Schramm 2007). Assessing the efficiency of such practices typically relies on stock assessments of biomass and maximum or optimum sustainable yield, both of which depend on assumed size to age relationships (Cotter et al. 2004; Lai et al. 2023). Reliance on these models may lead to incorrect assessments of size and assumed age structure of fish populations, which can result in erroneous fishing practice management and lead to downstream consequences including the loss of genetic diversity, population skews towards early maturation, trophic changes, and reduced lifetime reproductive output (Barneche et al. 2018). Further, there is an assumption that size‐age relationships are predicated on low variability, when in reality these vary greatly across space and time (Cao et al. 2020; Punt 2019) and with body condition (He et al. 2016).

Due to challenges in determining age and life stage of individuals, the demography of many fish populations is not commonly known, although this is fundamental to both understanding population and community dynamics and informing sustainable practices. Therefore, understanding population demography and underlying mechanisms contributing to maturation timing and lifespan (which are heavily influenced by environmental variation (Finigan and Marschall 2025; Fonseca and Cabral 2007)) is vital for predicting fish recruitment (Aguilar et al. 2023; Siepker and Michaletz 2013). Many commercially relevant species show sexual dimorphism with relation to growth, which should also be accounted for to improve stocking strategy estimates (Beamish et al. 2005). Recent studies have demonstrated that demographic and life history information can be inferred from epigenetic molecular modifications (Li et al. 2021; Vigneau and Borg 2021; Jonsson and Jonsson 2019; Kho and Ruzzante 2024), which do not change underlying nucleotide sequences but alter molecular structure and function (Al Aboud et al. 2018).

Patterns of DNA methylation (additions or reductions of methyl groups to carbon 5 position of cytosines from methyl transferases (Cheng 1995)) have been correlated with many physiological processes, including sexual function and determination (Ammerpohl et al. 2013; Piferrer 2013), aging and overall lifespan (Horvath 2013), and responses to environmental conditions (Kamstra et al. 2018; Mirbahai et al. 2011). These modifications occur most frequently in cytosines immediately neighbored by guanine nucleotides (Qi et al. 2015), which are commonly referred to as CpG loci. Methylation status at many CpG loci is thought to confer molecular integrity and maintain physiological function (Meng et al. 2015); however, altered methylation states may occur from molecular responses to lifetime stressors (Zannas et al. 2015). For example, global loss or erosion of methylation signals at CpG loci (hypo‐methylation) occurs with age (Pogribny and Vanyushin 2010), which is postulated to occur from the accumulated lifetime stress, degraded physiological function, and genomic instability that accelerates biological aging (Besselink et al. 2023). In fish, aging hypo‐methylation has been observed in island‐shore regions (Shimoda et al. 2014), as well as promoter and gene‐body regions (Rodríguez‐Casariego et al. 2020). Conversely, additions of methyl groups (hyper‐methylation) may also occur at specific loci (Yeh et al. 2017), which are associated with overall reductions in gene expression levels (Irizarry et al. 2009) and aging in humans (Dozmorov 2015; McKinney et al. 2015).

As a result of their high correlation, age‐associated CpG methylation patterns have been utilized to construct models (or epigenetic clocks) capable of accurately predicting biological age for a variety of model and non‐model organisms (Bors et al. 2021; Gardner et al. 2023; Hernandez et al. 2023; Horvath and Raj 2018; Shealy et al. 2025; Thompson et al. 2017; Arai and Inoue‐Murayama 2026). These epigenetic clocks are commonly constructed by evaluating aging signals of CpG loci from known age individuals through modelling techniques such as elastic net regression (Levy et al. 2025), which utilizes machine learning to estimate age from DNA methylation patterns. In addition to single CpG loci, Bertucci‐Richter et al. (2024) showed that coordinated gain or loss of methylation with age occurs in many genomic regions, which may also be used to predict aging. Highly accurate clocks for many fish species (Anastasiadi and Piferrer 2020; Bertucci et al. 2021; Mayne et al. 2020, 2023) have been constructed by training epigenetic aging models on approximately 70%–80% of the samples in the dataset, then using the remaining samples naïve to model construction as a testing set for evaluating clock predictions.

Traditionally, age estimations of LBc members have been obtained through otolith, scale, or fin (dorsal or pectoral) examination (Tyszko and Pritt 2017), although biases between reader agreements may lead to erroneous interpretations (Long and Fisher 2001; Sotola et al. 2014). While age estimation from otoliths yields improved accuracy compared to scale estimates (Tyszko and Pritt 2017), euthanasia is unfortunately required (Blackwell et al. 2019). Although the use of epigenetic patterns of fish species may hold promise for informing fisheries management practices (Piferrer and Anastasiadi 2023; Liu et al. 2025), epigenetic studies and associated responses to environmental variation and aging in the LBc have been limited (Chen et al. 2025; Liang et al. 2025; Weber et al. 2025). Recently, Weber et al. (2025) constructed an epigenetic model capable of accurately predicting age for Florida bass populations, thus demonstrating the applicability of this method for non‐lethal black bass demography assessments; however, the utility of such models for cross‐species assessments remains ambiguous.

In this study, we assessed age‐associated DNA methylation patterns from wild‐caught and hatchery fish and developed epigenetic age‐predictive models (using both single CpG loci and regional methylation patterns) capable of accurately predicting chronological age of LBc members. Simultaneously, we annotated physiological functions of age‐, sex‐, and location‐associated patterns. We predicted that global average DNA methylation levels should decrease with age, predicting genomic instability and matching prior study results of other piscine species (Zupkovitz et al. 2021; Bock et al. 2026), and that these patterns would vary among locations and with sexual differences. Additionally, as gain or loss of methylation at specific loci may influence aging patterns (Piferrer and Anastasiadi 2023; Bock et al. 2026), we predicted our final epigenetic clock models would contain loci becoming either hypo‐ or hyper‐methylated with age, indicative of directional genomic signals that accelerate aging processes. Characterization of methylome variability patterns in conjunction with epigenetic clock development will elevate our understanding of molecular physiology across the LBc and facilitate improved conservation and management practices for these trophy species.

## 2 Materials and Methods

### 2.1 Sample Collection and DNA Extraction

For this study, bass were collected from Tennessee (TN) and Texas (TX) water bodies. LBc individuals were collected from TN locations proximal to the Oak Ridge National Laboratory (ORNL) from September 26, 2023 to June 17, 2024 (Tennessee Wildlife Resources Agency collection permit: 2395) using an electrofishing boat in local reservoirs (Figure 1). The boat was equipped with a live well that fish were immediately placed in for recovery. All fish were collected using the lowest voltage and frequency necessary to efficiently capture fish without causing harm or unnecessary stress. Settings varied across sites based on water temperatures and conductivity. Fish with prior injuries were not retained for this study and were immediately released. Following all field collections, fish were brought back to ORNL. To include fingerlings in age analyses, live known‐age individuals (0.5 years of age) were sourced from Fish Wagon Hatchery on September 17, 2022. Following all collections, bass were euthanized by submersion in 20–40 mg/L of MS222, followed by cervical dislocation (ORNL Animal Care and Use Committee protocol: 0477). Bass from TX were collected from multiple locations, shown to previously be dominated by *M. salmoides* and hybrids with *M. nigricans* (Lutz‐Carrillo et al. 2023), by Texas Parks and Wildlife Department (TPWD) from February 27, 2000 to June 6, 2023 via boat‐mounted direct current electrofishing following TPWD—Inland Fisheries Division standard sampling procedures. Sampling was conducted along reservoir shorelines during daylight and collected bass were sacrificed via cervical dislocation. For each fish, fin clips were collected from the dorsal lobe of the caudal fin for epigenetic analysis and otolith samples were collected to obtain ages. This yielded a combined data set of 176 samples comprised of male and female LBc members spanning multiple age classes from 0.5 to 14 years from both TX and TN locations. DNA from fin clips (20–25 mg) was extracted using Qiagen DNeasy Blood and Tissue kits, and resulting concentrations were quantified using a Qubit fluorometer broad range DNA assay (Thermofisher Scientific). Individual ages for all LBc individuals were assessed using otolith ring estimation as described by Buckmeier and Howells (2003).

**FIGURE 1:**
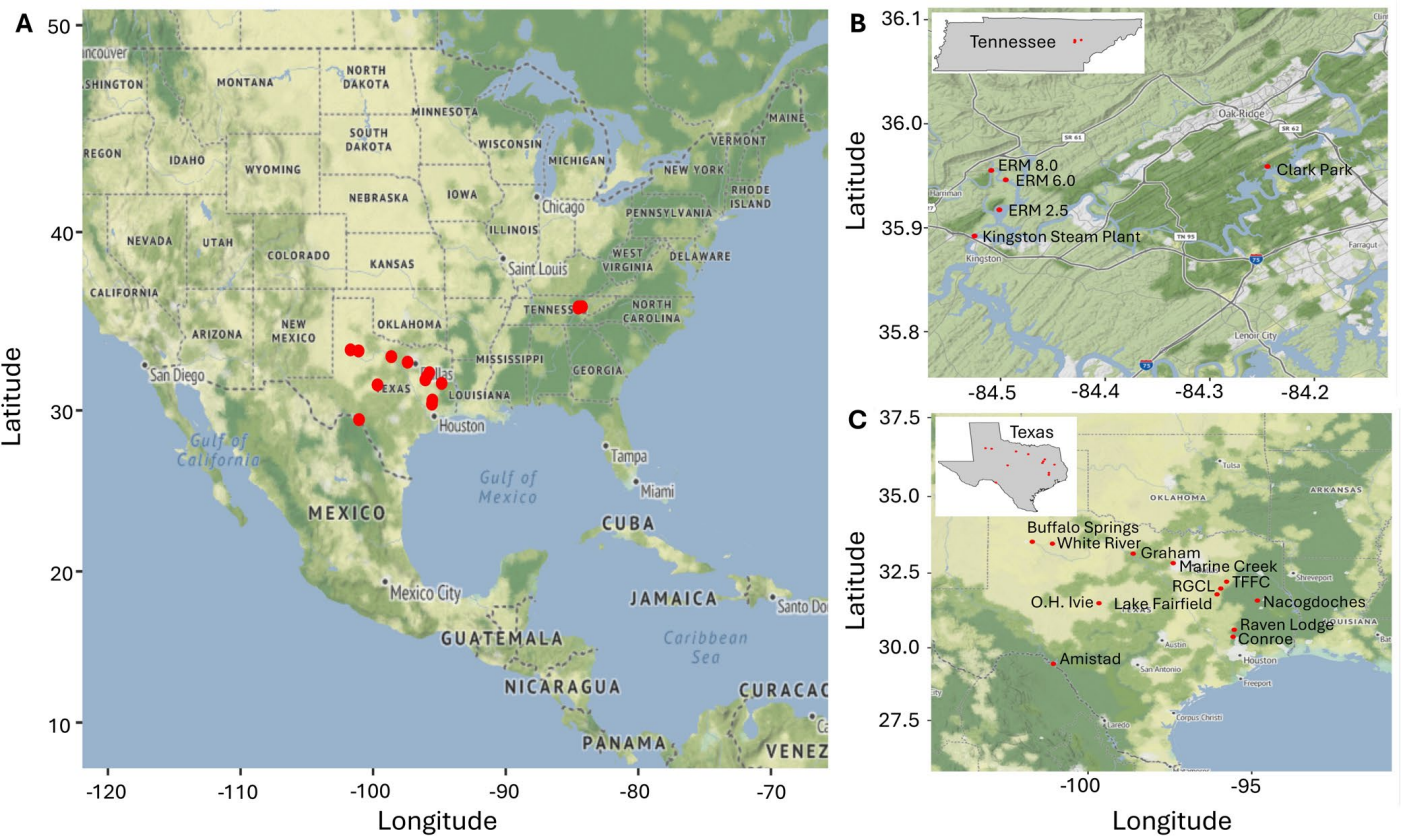
Sampling locations for LBc specimens. (A) Overview map of the Southeastern United States, illustrating the regional context of the sampling areas for members of the LBc. (B) Tennessee locations sampled for bass by Environmental Sciences Division staff at the Oak Ridge National Laboratory (ORNL) using via boat‐mounted direct current electrofishing. (C) Texas locations sampled for bass by Texas Parks and Wildlife Department (TPWD) via boat‐mounted direct current electrofishing. Map colors emphasize physical geography, with gradients of green representing vegetation and forest cover and blues representing hydrographic features.

### 2.2 Library Preparation and Sequencing

DNA methylation libraries for an initial subset of samples (*n* = 58) were prepared using 10 ng of DNA as starting quantity as directed from the protocol from extracted tissue DNA using the Zymo‐Seq Cell Free DNA WGBS (Zymo Research). Following this subset, libraries for remaining samples (*n* = 123) were prepared using the NEBNext Enzymatic Methyl‐seq kit from New England Biolabs (cat # E7120‐3), using 200 ng as starting material as directed by the protocol. Quality and quantity of all final libraries were assessed using an Agilent 4150 Tapestation D1000 assay, were diluted to 4 nM, and then pooled based on library preparation method. All libraries were subsequently sequenced using 150 bp paired‐end sequencing with an Illumina NovaSeq 6000 at the University of Tennessee Knoxville Genomics Core Facility.

### 2.3 Bioinformatic Analysis

Reads were quality checked and trimmed using FastQC (version v0.12.1) and TrimGalore! (version 0.6.10) to remove adapter sequences (stringency overlap = 1) and low‐quality reads (Phred score < 30). Paired reads that fell below 20 base pairs in length following trimming were discarded, although single reads from pairs above this threshold were retained as unpaired reads. Trimmed reads (paired and saved unpaired reads above 20 bp) were then aligned to a bisulfite‐converted index of the largemouth bass genome (ASM1485139v1) (Sun et al. 2021) using Bismark (version 0.24.0) (Krueger and Andrews 2011) allowing for one mismatch (option n‐1), and methylation percentages for alignments were performed (‐‐score‐min L,0,−0.6). This yielded an average of ~40 million reads with an alignment rate of ~68% for Zymo libraries, and an average of ~37 million reads with an alignment rate of ~71% for NEB libraries. Resulting bam files for each sample were sorted and merged using SAMtools (version 1.9) (Li et al. 2009).

### 2.4 CpG Methylation

Bam files were analyzed using the methylKit page (Akalin et al. 2012) in R (version 4.2.2). CpG methylated cytosines from each sample were retained using the read.context parameter in the processBismarkAln function. Only loci with 5× minimum read coverage were considered for analysis, and those above the top 99.95th percentile were discarded to account for potential PCR bias. Resulting cytosines from all samples were united using destrand = True option to merge reads from both strands of each CpG dinucleotide. For initial libraries prepared using Zymo‐Seq Cell Free DNA WGBS kit, only CpG loci covered in at least 70% of all samples were retained for analysis. For the larger number of libraries prepared using the NEBNext Enzymatic Methyl‐seq kit from New England Biolabs, this coverage requirement was relaxed to increase the likelihood of overlapping sites with initial Zymo libraries. As read yield and alignment rates from both library preparations were similar, methylation data for CpG loci common to both Zymo and NEB libraries were combined into one large dataset consisting of 4,289,413 CpG loci covered with at least 5× sequencing call depth. Duplicate samples from 6 individuals that had been sequenced across both libraries were removed from the dataset, as well as one individual whose otolith age was unavailable. The remaining samples were then assessed for completeness, and all samples with more than 80% of the total CpG loci missing were excluded from downstream analyses. All CpG loci that were present in at least 70% of the remaining samples were retained into a final dataset consisting of 156 bass (Data S1) with 225,261 CpG loci.

### 2.5 Patterns of CpG Methylation Variability

We first assessed contributions of age, sex, and location to methylome variation by computing Gower's dissimilarity indices (weighted distance measures that are used to compare similarity of data observations which have been used previously to evaluate DNA methylation data (Rodríguez‐Casariego et al. 2020)) from CpG methylation matrices from the final dataset using the vegdist function from the vegan package in R (Dixon 2003). To account for any missing data prior to this analysis, we first performed imputation on samples missing data for these sites using a *K*‐nearest neighbor (KNN) approach using the impute package (Hastie et al. 2017) in R with the following settings: *k* = 3, rowmax = 0.5, colmax = 0.8, maxp = 1500. This approach is used to fill in missing data by assigning values derived from the most similar non‐missing data (Zhang 2016), in this case, the next three most similar bass samples based on non‐missing methylation loci. Resulting dissimilarity indices for methylation data were visualized by conducting non‐metric multidimensional scaling (NMDS) using vegan's metaMDS function with 999 iterations and 500 random starts. Dimension numbers (*k*) were initially set to 3 but were adjusted if necessary to ensure minimization of resulting stress levels. Effects of age, sex, and location were fitted as vectors in resulting ordinations, and the percentage of methylation at all loci from each individual from the final dataset was evaluated for association with these variables using the anosim function in R.

Global average methylation was calculated as the average methylation percent for all retained and imputed CpG loci in the final dataset. This was done as global methylation has been shown to be associated with physiological changes with senescence (Bellizzi et al. 2012; Fang et al. 2013), sex (Liu et al. 2010), and response to environmental conditions (Martin and Fry 2018). As the global average methylation values are bound between 0 and 1 (Marttila et al. 2015), beta regression analysis using a logit link function was used with sex and location as factors and age as a covariate using the betareg package in R to determine the effects of sex, age, or sampling location on global average methylation patterns.

To obtain potential methylation patterns and pathways contributing to physiological differences from age, sex, and location in members of the LBc, the coverage requirement for our final dataset was relaxed to include all loci present in at least 60% of the samples, which yielded an exploratory dataset consisting of an additional 1,753,083 sites for a combined total of 1,978,344 CpG loci. Differential methylation analysis was subsequently performed on these loci using the DMLfit.multiFactor function from the DSS package in R (Feng and Wu 2019). This fitted a beta‐binomial model to the methylation data using age as a covariate, and sex and location as factors. Interaction terms among these variables were also included, resulting in the final formula (methylation ~ age + sex + location + age*sex + age*location + sex*location + age*sex*location). Wald tests were subsequently performed within the DMLfit.multiFactor function for all loci for each model term. Multiple‐testing correction was performed on the resulting *p*‐values using Benjamini and Hochberg's FDR adjustment (Benjamini and Hochberg 1995) set to 0.05, and all loci with FDR values < 0.05 for each term were considered to be differentially methylated (Lawrence et al. 2013). Cytosine methylation in different genomic regions, with different CpG densities (islands, shores, shelves, open seas) and gene proximity (promoters, introns, and exons), impacts molecular and downstream physiological function (Garcia‐Prieto et al. 2022; Han et al. 2008; Lokk et al. 2014; Sae‐Lee et al. 2018; Anastasiadi et al. 2018; Lev Maor et al. 2015). To assess physiological changes from methylation patterns in the LBc associated with size‐at‐age, differentially methylated CpG loci were first annotated according to CpG density and then according to their gene proximity (introns, exons, or intergenic regions) using the genomic ranges package in R.

CpG islands, regions of high CpG density, have commonly been associated with transcription and gene promoter regions (Antequera 2003; Ioshikhes and Zhang 2000). The terms shores and shelves refer to CpG loci further away from islands (2000 and 4000 bp flanking either side of identified CpG islands, respectively), which are also implicated with controlling levels of gene expression (Irizarry et al. 2009; Long et al. 2017; Visone et al. 2019). All remaining CpG loci are defined as belonging to open sea regions, the remaining vast isolated regions of the genome which are the most numerous (Moran et al. 2016; Carmona et al. 2017) and whose methylation status is thought to be associated with genomic structure and stability (Rodriguez et al. 2006). CpG islands for the LBc were annotated using the algorithm developed by Takai & Jones (Takai and Jones 2002), which utilizes a python script (TaJoCGI.py) to identify regions ≥ 500 bp with GC densities ≥ 0.55. Shores were defined as the 2000 bp flanking either side of identified CpG islands, shelves as another 2000 bp outward, and all remaining CpG loci were defined as open sea regions. Gene annotations (introns, exons, promoters) were obtained with the GenomicFeatures package in R (Lawrence et al. 2013) using the largemouth bass genome gff file.

To assess whether observed genomic methylation patterns (CpG density and gene context proximity for each model term) occur with significance or from random chance, proportions of CpG loci with significant FDR values from each model term (hyper‐ and hypo‐methylated) found to overlap with these regions were compared with proportions of all final dataset loci falling in these same regions using exact binomial tests. Proportions of hyper‐methylated loci were also compared with hypo‐methylated proportions for each term. Significance of binomial test comparisons indicates the loci that gain or lose methylation for each term do so in a directed manner, rather than by random chance. Additionally, functional pathway annotations for hypo‐ and hyper‐methylated loci were identified with KEGG overrepresentation enrichment analysis using the clusterprofiler package (Xu et al. 2024) with the following parameters: (pvalueCutoff = 0.05, pAdjustMethod = “BH”, minGSSize = 10, maxGSSize = 500, qvalueCutoff = 0.2).

### 2.6 Construction of Initial Epigenetic Age Models

#### 2.6.1 CpG Age Models

Epigenetic models for predicting age of members of the LBc were constructed by randomly assigning 80% of our samples into a training set (124 individuals) for model construction, and the remaining 20% (32 individuals) into a testing data set naïve to the model construction for performance evaluation. To ensure there was no influence of the test set on model construction, imputation was performed separately as described previously within each dataset to remove any missing values. Next, loci with zero or near zero variance in the training set were removed, similar to methods by Anastasiadi et al. (2026). Age predictive models were then constructed from the remaining loci (*n* = 81,071) in the training set using elastic net modelling (alpha = 0.5, family = gaussian) from the glmnet package in R (Friedman et al. 2021) which identifies loci capable of accurately predicting age by assigning penalties to individual coefficients of model‐selected CpG‐methylated cytosines, a method previously used in epigenetic aging studies (Robeck et al. 2023). Previous studies have shown increased accuracy when natural log transforming age prior to model construction (Weber et al. 2025; Anastasiadi et al. 2026); therefore, our clock was constructed twice using either natural log‐transformed age (treated as log(age + 0.1) similar to (Anastasiadi et al. 2026)) or non‐transformed age as the response variable, and the model yielding the best performance when evaluating the test set was then selected as the final model. Errors resulting from the final model predictions were then assessed for association with age, sex, and location using the lm function in R. To ensure aging signals of the selected loci used in the final CpG model were not influenced by sex or location, FDR values from our DSS model for age*sex, age*location, and age*sex*location terms for each chosen loci were evaluated for significance (FDR < 0.05). Chosen sites were also annotated according to proximity to regions of CpG density, gene context, and for KEGG term enrichment using clusterprofiler.

#### 2.6.2 Regional Methylation

To assess the utility of larger genomic windows in predicting age across the LBc, bam files from prepared libraries in the final dataset were first converted to human readable SAM .txt files using samtools view from Samtools version 1.9. Methylation call strings from each read were then extracted in R and were subsequently mapped to 200 bp windows of the largemouth bass genome, similar to methods used by Bertucci‐Richter et al. (2024). This was performed by first binning the genome into 200 bp windows using the ‘makewindows’ function from Bedtools (v2.26.0) and then using the Bedtools ‘map’ function to average the per‐read methylation percent for all reads. To ensure reads were kept from being represented in more than one region, each read was uniquely assigned to an individual window when > 51% of that read mapped to a specific window. Average methylation across these 200 bp windows was calculated using the mean proportion of methylated cytosines within each region. Similar to the final CpG dataset, only regions covered in at least 5× sequencing depth and 70% of all were retained for analysis. This yielded a final data set of 548,045 200 bp regions.

Regional methylation patterns were used to predict age in members of the LBc following similar methods as for single CpG loci analyses, using 514,943 regions following the removal of zero or near zero variance regions. Performance between regional models using either age or natural log‐transformed age was evaluated, with accuracy of test set predictions being used to select the final regional model. To allow comparison between models, the same set of individuals used in the CpG loci training set were used to train the 200 bp region elastic net model, which was then evaluated by predicting ages of the test set. Associations with sex and location for regions in this model were assessed by evaluating FDR‐corrected *p* values obtained from linear regression with methylation percentage as the independent variable and age, sex, location, and all interaction terms as dependent variables using the lm function in R. Regions with significant (FDR < 0.05) values for age*sex, age*location, and age*sex*location terms would indicate associations with sex and location on aging signals in the LBc.

## 3 Results

### 3.1 Factors Associated With CpG Methylation Variability

#### 3.1.1 Non‐Dimensional Scaling

From the combined Zymo and NEB data, retained CpG sites were grouped with non‐dimensional scaling with *k* = 5 dimension values, yielding a stress value of 0.11 (non‐metric fit *R* ^2^ = 0.988, linear fit *R* ^2^ = 0.972) (Figure 2A). Although both age and sex significantly influenced dimensional variation (*p* < 0.05, 999 permutations, Gower's dissimilarity), separation was greater for age (Global *R* = 0.29) compared to sex (Global *R* = 0.03). There was little effect of location on non‐dimensional scaling patterns (ANOSIM: Global *R* = 0.03, *p* = 0.21) (Figure 2B).

**FIGURE 2:**
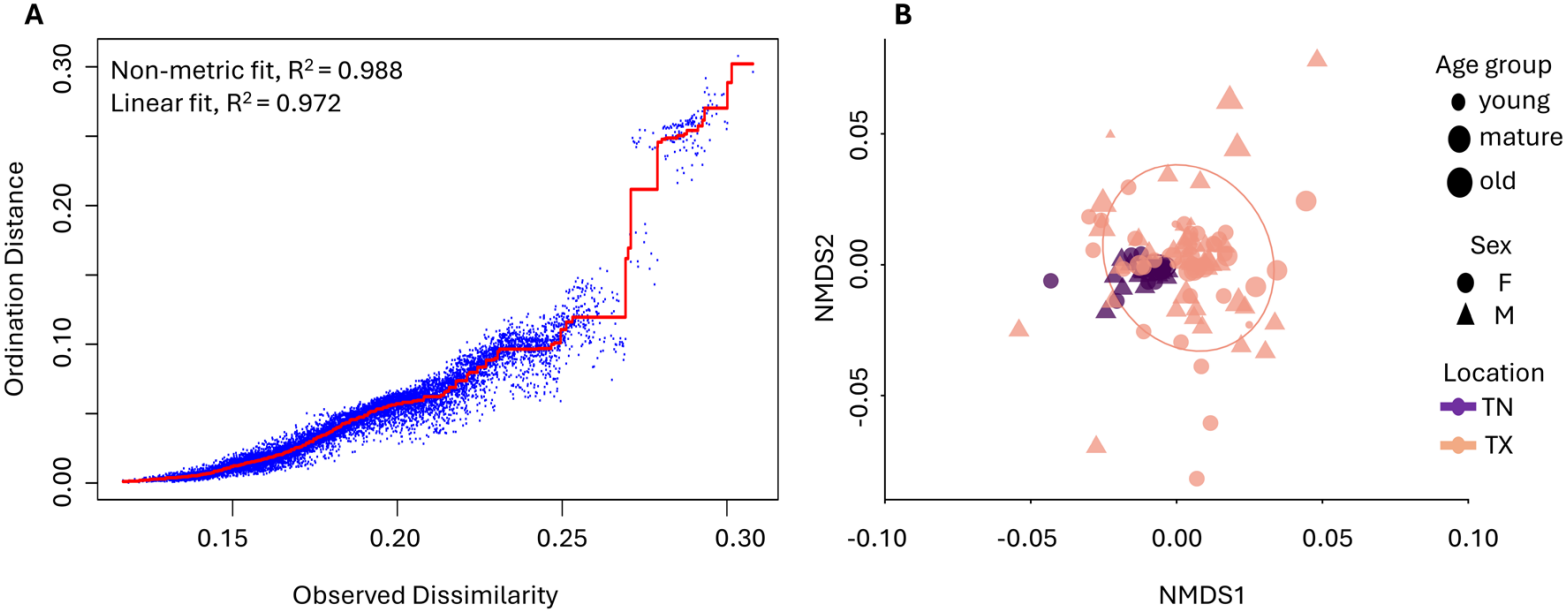
Overall variability in largemouth bass methylome. (A) Stress plot visualizing goodness of fit of matrix methylation data for CpG loci to distances in ordination space. Ordination distances between samples increased as dissimilarities in methylation increased. (B) NMDS plot showing how dissimilarities in methylation data occur based on association with age, sex, and location across non‐dimensional scales (NMDS) 1 and 2. Each point represents an individual sample, with the distance between points corresponding to CpG methylation value similarity. Samples are color‐coded based on sampling location (TX indicating bass collected from Texas, and TN representing bass collected from Tennessee), with ellipses representing distribution spread among sampling locations. Shapes denote sex (circles are females and triangles are males) and shape size corresponds to bass age (small size indicates young bass (< 2 years), medium size indicates sexually mature fish (age 2–8), and large sizes indicate older fish (age 9+)).

#### 3.1.2 Age

Global average methylation significantly decreased with age according to the beta regression model which included age as a covariate and sex and location as factors (*B* = −0.018, SE = 0.005, *z* = −3.465, *p* < 0.001). The precision parameter was estimated at *φ* = 158.82 (SE = 17.96), and the overall model pseudo‐*R* ^2^ was 0.32 (Figure 3A). According to Benjamini and Hochberg's FDR corrections of Wald test results within the DMLfit.multiFactor function, methylation levels of 1314 loci changed significantly with age (FDR < 0.05), of which 821 (62.5%) were hypo‐methylated (i.e., lost methylation), while 493 (37.5%) were hyper‐methylated (i.e., gained methylation). Annotations for loci differentially methylated with age revealed significant overlaps of both regions of CpG density and gene regions according to exact binomial tests when comparing these loci with all background CpG sites (the 1,978,344 total CpG loci of the large dataset) (Figure 3B,C). Additional binomial tests were performed by comparing hypo‐ and hyper‐methylated loci probabilities (Raw data and values of exact binomial tests for overlaps relative to CpG density and gene proximity for differentially methylated loci according to age, sex, and location can be found in Data S2). KEGG overrepresentation enrichment analysis revealed 4 GO terms (FDR < 0.05) associated with loci significant for age (Figure 3D).

**FIGURE 3:**
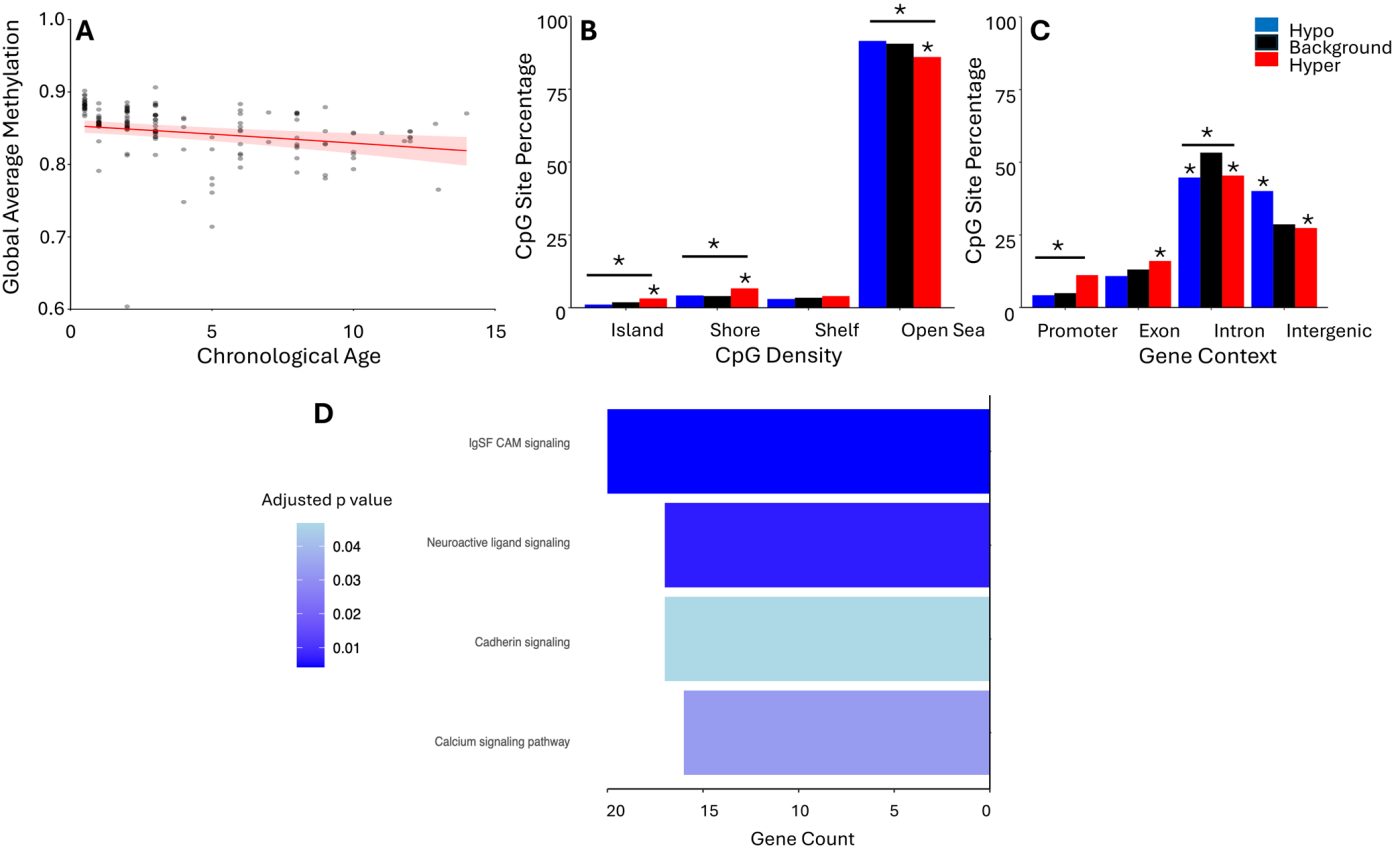
Effect of age on the largemouth bass methylome. (A) Global average methylation decreased with age (*p* < 0.05). (B) Overlaps of differentially methylated loci (FDR < 0.05) from aging with regions of differing CpG densities. (C) Overlaps of differentially methylated loci from aging with gene regions. Asterisks (*) indicate significant enrichment or depletions of loci in these regions relative to background sites for hypo‐ (blue) and hyper‐methylated loci (red) with age, with bars indicating significant differences between hypo‐ and hyper‐methylated loci. (D) Enriched pathways for loci hypo‐methylated with age in the LBc.

#### 3.1.3 Sex

Global average methylation showed a trend of being higher in female compared to male bass, although this was not significant according to the beta regression model which included age as a covariate and sex as a factor (*B* = 0.068, SE = 0.035, *z* = 1.918, *p* = 0.06) (Figure 4A). There were 64 loci that differed significantly between males and females, with 56 (87.5%) of these loci having higher methylation values for females and 8 (12.5%) having higher methylation values for males. Similar to loci differentially methylated with age, annotations for loci differentially methylated between males and females revealed specific overlapping patterns with CpG density and gene regions according to exact binomial tests (Figure 4B,C). There were no KEGG terms associated with sex.

**FIGURE 4:**
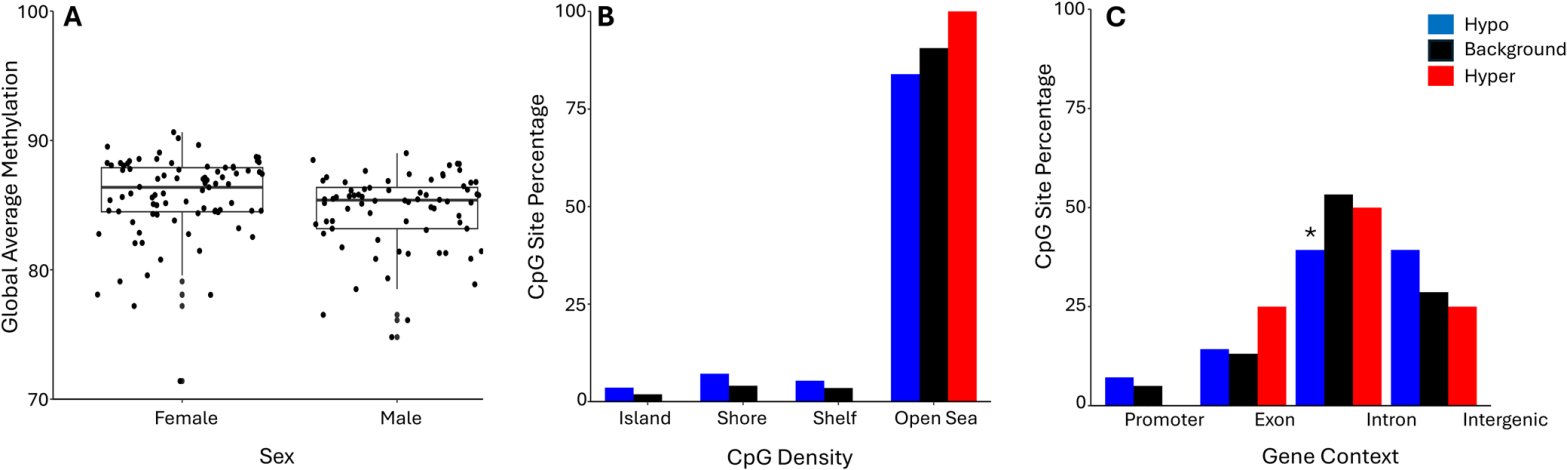
Effect of sex on the largemouth bass methylome. (A) Global average methylation was slightly elevated in female compared to male bass (*p* = 0.07). (B) Overlaps of differentially methylated loci (FDR < 0.05) between males and females with regions of differing CpG densities. (C) Overlaps of differentially methylated loci between males and females with gene regions. Asterisks (*) indicate significant enrichment or depletions of loci in these regions relative to background sites for loci hypo‐methylated (blue) and hyper‐methylated (red) in males compared to female bass, with bars indicating significant differences between hypo‐ and hyper‐methylated loci.

#### 3.1.4 Location

Global average methylation was significantly elevated in bass collected from TN compared to those from TX according to the linear regression model which included age as a covariate and sex and location as factors (*B* = 0.19, SE = 0.04, *z* = 4.87, *p* < 0.001) (Figure 5A). When evaluating the effect of sampling location on the methylome, there were 55,265 loci whose methylation status differed significantly between TX and TN bass, of which 10,409 (18.83%) showed higher methylation levels for TX bass, while 44,856 (81.17%) had higher methylation levels for TN bass. There were specific overlapping patterns of differentially methylated loci between TX and TN with both regions of CpG density and gene regions (Figure 5B,C), and there were 57 KEGG terms associated with location (Figure 5D).

**FIGURE 5:**
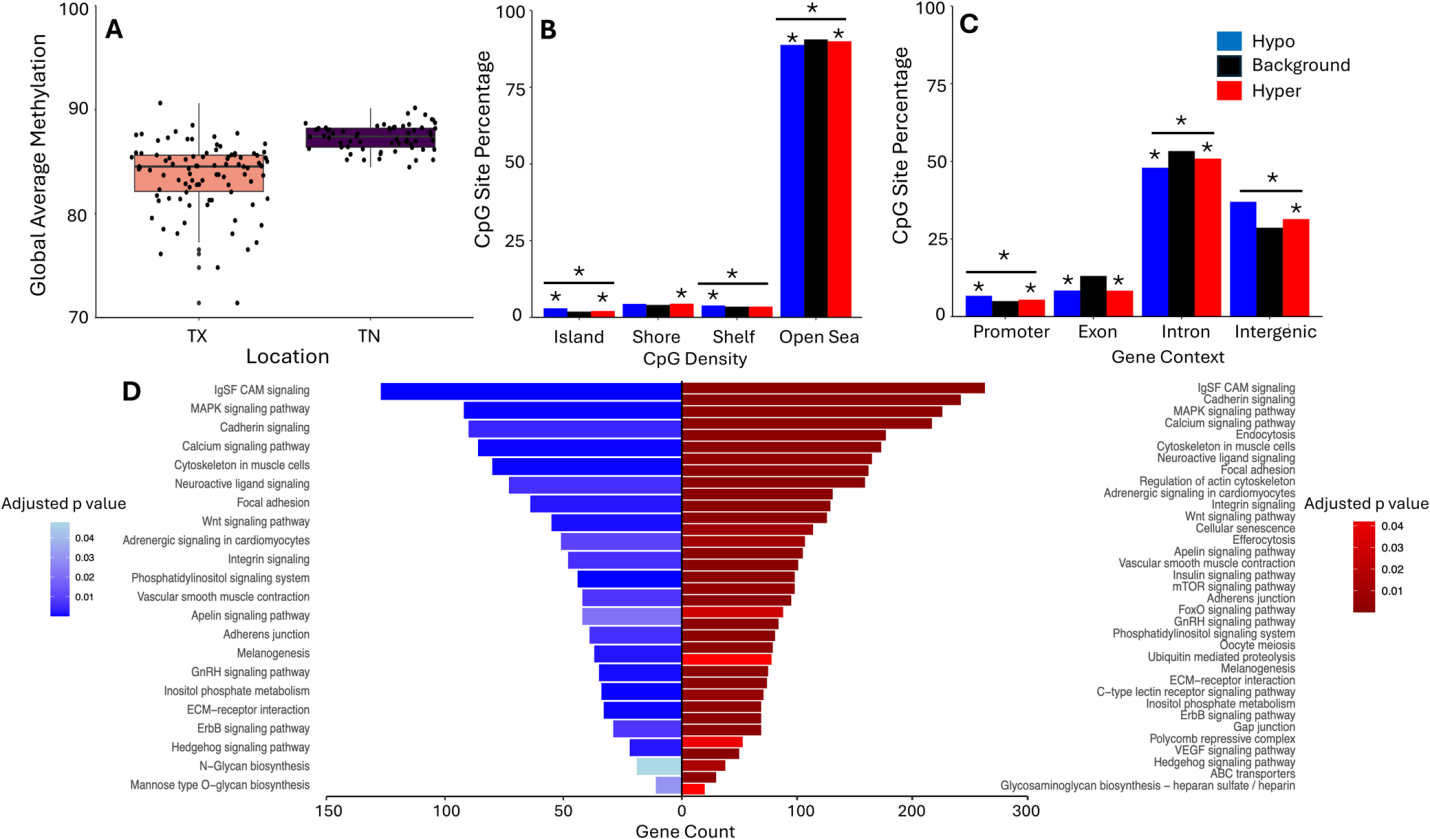
Effect of sampling location on the largemouth bass methylome. (A) Global average methylation was higher for TN bass compared to those from TX (*p* < 0.05). (B) Overlaps of differentially methylated loci (FDR < 0.05) in TN bass compared to TX with regions of differing CpG densities. (C) Overlaps of differentially methylated loci between TX and TN bass with gene regions. Asterisks (*) indicate significant enrichment or depletions of loci in these regions relative to background sites for loci hypo‐methylated (blue) and hyper‐methylated (red) in TN bass compared to TX bass, with bars indicating significant differences between hypo‐ and hyper‐methylated loci. (D) Enriched pathways for hypo‐ and hyper‐methylated loci for TN compared to TX bass.

#### 3.1.5 Interactions Between Age, Sex, and Location

Differential methylation analysis also revealed loci affected by interactions between age, sex, and location. Interactions between age and sex indicate that the pattern for some loci that undergo changes in methylation levels with age differs in male compared to female bass. For example, of 2968 loci whose methylation level changed with age, the absolute value of the changes for 2642 (89.01%) loci was lower for males compared to females, with this pattern being reversed for 326 (10.99%) of these loci.

Interactions between age and location indicate aging patterns differ between TX and TN LBc members. For example, there were 245 loci whose absolute change in methylation levels with age differed according to location (TX vs. TN), with 212 (86.53%) showing larger absolute changes for TX bass, while 33 (13.47%) of these loci showed greater absolute changes for TN bass relative to age. The interaction between sex and location indicates that absolute differences between male and female bass change depending on location, indicated by 64 loci that differed significantly for this interaction term. From these, methylation level differences of 56 loci (87.5%) were greater between sexes of TX bass compared to those from TN, while 8 (12.5%) showed the opposite pattern.

The final term used in the DSS model (Age*Sex*Location) indicates the presence of loci whose differences in absolute methylation change between male and female bass with age is associated with location. For example, there were 18 loci whose methylation level of change between sexes with age differed between TX and TN, as 8 (44.44%) showed greater differences between males and females with age for TX bass, and 10 (55.56%) showed greater differences between males and females with age for TN bass (for overlaps and binomial test results of loci from interaction terms, see Figures S1A,B–S4A,B).

There were 4 terms associated with the interaction of age with sex, “IgSF CAM signaling”, “MAPK signaling”, “Phosphatidylinositol signaling system”, and “GnRH signaling”. There were no terms associated with the interaction of age with location, and one term associated with the interaction of sex with location, “vascular smooth muscle contraction”. There were no terms associated with the term age*sex*location. Full annotation data for loci found to be differentially methylated for each model term can be found in Data [Link], [Link], [Link], [Link], [Link], [Link], [Link].

#### 3.1.6 Construction of Initial Epigenetic Age Models

The final CpG model constructed from our training set used 136 loci to predict age (non‐transformed) in our training set with an *R* ^2^ value of 0.99 (Figure 6A). When using this model to predict ages for the test set, this model performed with an *R* ^2^ value of 0.78 and an MAE of 0.99 years (8.4% of the age range for test set individuals) (Figure 6B). All chosen loci used to predict age in this model had FDR values > 0.05 according to each of the interaction terms with age from our DSS model, indicating no association with sex or location. Errors of clock predictions compared to otolith ages did not vary with age (*B* = 0.03, *t* = 0.47, *p* > 0.05), sex (*B* = 0.20, *t* = 0.60, *p* > 0.05), or location (*B* = 0.20, *t* = 0.55, *p* > 0.05). There were no GO terms associated with clock‐selected loci (Data S10).

**FIGURE 6:**
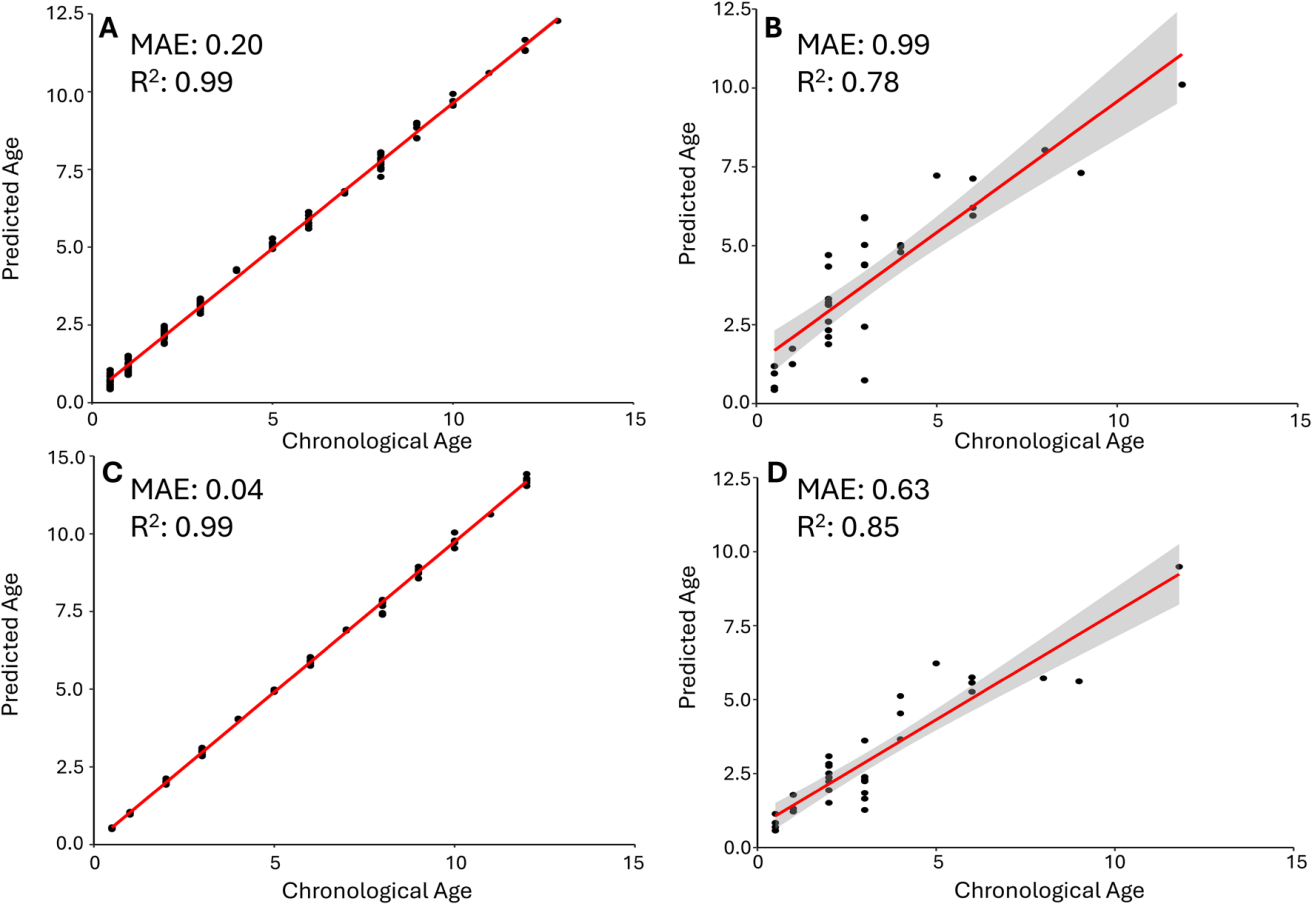
Performance of epigenetic clocks for predicting age in largemouth bass. (A) Performance of the elastic net regression model to predict age from single CpG loci trained on 124 bass from TX and TN (80% of the total data set) with values representing estimated ages and errors (reported as the median absolute error (MAE)) for the training data set. (B) Predicted compared to chronological ages in testing set (*n* = 32) from elastic net regularized regression approach (test set is standardized across remaining clocks for comparison). (C) Performance of the elastic net regression model to predict natural log‐transformed age from 200 bp regions comprised of CpG loci that gain or lose methylation similarly with age trained on the same 124 bass (80% of the total data set) from TX and TN as in the single CpG loci model. Values represent back‐transformed estimated ages and errors (reported as the median absolute error (MAE)) for the training data set. (D) Predicted compared to chronological ages from the 200 bp region model after back‐transforming estimates using the same testing set (*n* = 32) as in the single CpG loci model.

The final regional model (constructed using natural log‐transformed age) used 143 regions to predict age. When back‐transforming age estimates, this model performed similarly as the final CpG loci model, predicting ages in the training set with an *R* ^2^ value of 0.99 (Figure 6C). When using this model to predict ages for the test set, this model performed with an *R* ^2^ value of 0.85 and an MAE of 0.63 years (5.3% of the age range for test set individuals) (Figure 6D). The chosen regions for this model (Data S11) all had FDR values above 0.05 according to sex and location interaction terms with age, indicating methylation changes in model regions were not associated with sex or location (*p* > 0.05). Compared to the CpG model, predicted errors increased with age (*B* = 0.13, *t* = 2.36, *p* < 0.05) but did not differ based on sex (*B* = 0.39, *t* = 1.39, *p* > 0.05) or location (*B* = 0.11, *t* = 0.43, *p* > 0.05) (Data S12).

## 4 Discussion

Epigenetic aging studies with non‐lethal methods stand to advance fisheries science, natural resources management, and conservation of recreational and commercially important species. This approach can improve our understanding of ecosystem resilience, which is critical in an ever‐changing world. Here, we demonstrate that epigenetic clocks can be successfully constructed for closely related species from non‐lethal methods, which supports other work suggesting epigenetic aging signals may be conserved (Lu et al. 2023), potentially across piscine species (Chevrier et al. 2026). These models were used to determine the age of LBc members, commercially and recreationally important fisheries species, despite the influences of sex and location on methylome patterns.

Many factors contribute to DNA methylation variability (Rodríguez‐Casariego et al. 2020). Using combinations of NMDS and global methylation assessment, we identify that age, sex, and location are all significantly associated with dynamic methylation in the LBc, similar to results of other studies (Anastasiadi et al. 2026; Vanyushin et al. 2009; Grant et al. 2022; Keller et al. 2016; Varriale and Bernardi 2006). Many regions in the methylome often become hypo‐methylated with age as a consequence of epigenetic drift (Jones et al. 2015), resulting in abnormal gene expression regulation and physiological functioning (Unnikrishnan et al. 2018). Sex‐specific DNA methylation patterns also exist among many species (Bock et al. 2022; Chatterjee et al. 2016; Zhou et al. 2019), including fish (Valdivieso et al. 2023; Wang et al. 2025; Li et al. 2022; Navarro‐Martín et al. 2011), which have been associated with differential expression of genes and activity of physiological pathways (Chatterjee et al. 2016). A main example of this is the patterns of sexual dimorphic growth exhibited by many species (Parker 1992), which are associated with epigenetic aging rates during developmental windows (Strand et al. 2025). For example, the methylation status of select CpG loci has been attributed to patterns of sex‐specific aging in cownose ray (Weber, Wyffels, et al. 2024) as well as scorpion fish (Weber, Fields, et al. 2024). Decreased global methylation levels have also been observed previously among male fish (Sun et al. 2016). This may occur from increased demands for, and physiological differences associated with, masculinizing gene expression (Rubin et al. 2020). As global average methylation was shown to significantly decrease with age in the LBc, while showing a trend of being diminished in males compared to females, further assessment of gene expression and growth patterns associated with the regions identified as being differentially methylated with respect to age or sex, respectively, is of further interest.

In addition to age and sex‐related changes, climate and other environmental differences have been shown to affect DNA methylation patterns in fish (Keller et al. 2016). For example, populations of Atlantic cod from differing elevations and latitudes have been observed with differing global methylation profiles (Varriale and Bernardi 2006), referred to as a “latitudinal cline” in methylation by Anastasiadi et al. (2026). Shifts in environmental conditions, such as average temperatures and photoperiod, along such latitudinal gradients are correlated with differing methylation levels (Metzger and Schulte 2017; Jiang et al. 2026; Vinagre et al. 2009), which could influence this phenomenon. The global methylation variability observed in this study provides supporting evidence to latitudinal clines in DNA methylation observed previously (Anastasiadi et al. 2026; Liu et al. 2022), although the specific conditions associated with these population differences require additional assessment.

Regarding genetic pathways, loci differentially methylated for our DSS model terms showed overlaps with many genes of differing physiological function, which varied with each model term. This indicates dynamic methylome patterns across the LBc occur with age, are influenced by sex, and are impacted by environmental conditions, thus corroborating results from other studies showing the influence of latitude on life history patterns of fish (Heibo et al. 2005; Slaughter et al. 2008). Activity of IgSF CAM signaling, a pathway enriched for many of our model terms, is associated with neurodegeneration (Wang et al. 2026) which commonly occurs with age in many organisms (de Bakker and Valenzano 2023). Significant enrichment of this pathway for loci differentially methylated with age, as well as with interactions of sex and location, could indicate potential for LBc to experience neurodegenerative and aberrant behaviors with age, the degree of which differs with sex and thermal regimes associated with differing latitudes. This has been observed in other piscine species, as zebrafish have shown reduced locomotor activity with age, in addition to differential behaviors between sexes, such as depth preference in females and reduced thigmotaxis in males (Hudock and Kenney 2024). Water temperatures significantly affect activity of IgSM CAM signaling in fish (Vega et al. 2026), which are also associated with aging and behavioral differences. For example, lower temperatures are associated with delayed onset of locomotion and behavioral deficits associated with age in turquoise killifish (Valenzano et al. 2006). Investigation of the pathways identified here as being associated with aging in the LBc is therefore warranted. Although there were no enriched Go terms from our clock loci, several of these model‐selected sites showed overlaps with genes coding for immune and hormone receptors (Chang et al. 1988; Patel and Brewer 2008; Masternak and Bartke 2012; Renshaw et al. 2002), which may indicate decline in immune and endocrine function associated with disease and inflammation occur in the LBc with age (Ren and Yu 2022). Further research assessing associations between the locations of loci (overlap with promoters, exons, introns) and resulting downstream expression patterns is needed to fully explore the links between epigenetic signals and these pathways across the LBc.

The primary purpose of epigenetic model construction for fish is the development of an accurate nondestructive aging method. Non‐lethal age prediction with improved accuracy over conventional methods is critical to sustainable fisheries management and conservation. Performance from our models is comparable to those from other epigenetic aging studies (Gardner et al. 2023; Giannuzzi et al. 2024; Shealy et al. 2025; Weber, Fields, et al. 2024), with the number of sites in our clocks ranging from 136 loci to 143 regions respectively, which overlaps with the range of sites used in other piscine studies (Weber, Fields, et al. 2024; Weber et al. 2025; Anastasiadi et al. 2026). These clocks should therefore lend themselves well to development of downstream targeted assays for evaluating age. It has been postulated that minimum sample size for reliable epigenetic clock construction is 70 individuals, with 134 being more ideal (Mayne et al. 2021). Although slightly less than this optimal value, our training set size of 124 for both models fell within this range. Additionally, prediction errors in our testing set did not vary with either sex or location. Similar to prior studies (Weber, Fields, et al. 2024), our CpG model errors did not increase when predicting ages for older individuals; therefore, allowing for distinction of multiple age classes using non‐destructive methods.

Variability influenced by molecular library preparation and sequencing approach may limit the applicability of single CpG loci selection for age predictions (Bertucci‐Richter et al. 2024). Expanding site selection from single loci with methylation gain or loss to regional methylation change with age should allow for increased likelihood of usable sites for downstream aging or cross‐study applications. Our regional model showed improved performance compared to our CpG loci model, matching results of Simpson et al. (2023); however, this came with a slight tradeoff as prediction errors from our final regional model did increase with older individuals, a phenomenon that has been observed in many other aging studies (Gardner et al. 2023; Bock et al. 2026; Anastasiadi et al. 2026; Weber, Fields, et al. 2024) that is postulated to occur due to age‐specific rates of methylation change (Weber, Fields, et al. 2024; Horvath and Raj 2018). Extension of clock sites from single loci to regions could have captured such patterns in the LBc, indicated by the improved performance of our regional model when natural log‐transforming age, which has been shown to improve the modeling of non‐linear patterns (Simpson and Chandra 2021); however, as our training calibration dataset contained higher numbers of younger individuals, this pattern could also be associated with under‐representation of older individuals in our training dataset, which has been observed to influence epigenetic clock performance (Teschendorff and Horvath 2025).

Conventional non‐lethal aging methods for black bass have involved counting annuli rings from scales, as well as dorsal and anal spines (Blackwell et al. 2019; Werner et al. 2025). Although scale age estimates have been shown to be somewhat reliable for younger individuals (Blackwell et al. 2019), scale, dorsal, and anal spine predictions generally underestimate ages for older bass (Werner et al. 2025), and high variation may exist between readers. Additionally, estimates from traditional nonlethal methods are generally capable of predicting ages up to approximately 9 years (Blackwell et al. 2019; Werner et al. 2025; Klein et al. 2017), while our CpG and regional models predicted age up to 10.1 and 9.5 years, respectively. Therefore, epigenetic age assessment may be more reliable for older age classes compared to other nonlethal methods. From a logistics perspective, the costs of initial whole genome methylation and sequencing studies are financially costly ($20‐30 K); however, follow‐on study costs using targeted methods (Giannuzzi et al. 2024) developed from the initial sequencing investment are greatly reduced. For example, methods involving minion sequencing of PCR amplified target regions are much lower (Qi et al. 2024), with average costs per sample ranging from $7 to $10 per sample.

Application of targeted approaches with the CpG loci and regions of our clocks in further assay development could improve age demography assessments across the LBc, particularly for older individuals, with comparable cost involved with reading annuli when personnel time and fees are considered. Provision of this tool would allow for improved nonbiased and nonlethal age predictions for older age classes, which will likely aid in estimates of population recruitment, age class mortality, and reproductive success, while better informing harvest regulations and stocking procedures for LBc fisheries (Feltz and Catalano 2017; Tyszko and Pritt 2017; Post et al. 1998; Lorenzoni et al. 2002). By annotating potential factors such as age, sex, and location associations with methylome variability across the LBc, we were also able to identify epigenetic signals associated with life history patterns and molecular signals. We developed robust models for improved nonlethal age predictions across the LBc that were not affected by these associations and performed similarly to other models (Weber et al. 2025; Anastasiadi et al. 2026; Anastasiadi and Piferrer 2020), which can better inform black bass management practices in downstream applications.

## Funding

This work was supported by Oak Ridge National Laboratory’s Laboratory Directed Research and Development Grant (11077), and the USFWS Federal Aid in Sport Fish Restoration Grant (TX F‐231‐R‐5).

## Conflicts of Interest

The authors declare no conflicts of interest.

## Supporting information


**Data S1:** LBc metadata. Metadata listing otolith ages, location of origin, sex, specific capture location, and GPS coordinates of bass belonging to the LBc used to assess factors influencing the methylome variation and construct nonlethal epigenetic clocks.

**Data S2:** Overlaps of differentially methylated CpG loci with differing regions of CpG density and gene regions according to age, sex, and location. Background loci are in bold, with symbols: (−) and (+) indicating loss or gain in methylation.
**Figure S1:** Interactions between age and sex across the LBc methylome. (A) Overlaps of loci whose methylation status changes differ between males and females relative to age (FDR < 0.05) with regions of differing CpG densities. (B) Overlaps of loci whose methylation status changes differ between males and females relative to age (FDR < 0.05) with gene regions. Asterisks (*) indicate significant enrichment or depletions of loci in these regions relative to background sites for loci whose changes are lower (blue) or higher for males (red) compared to females, with bars indicating significant differences between loci. (C) Enriched pathways for loci whose methylation changes are lower for males (blue) with age compared to females.
**Figure S2:** Interactions between age and location across the LBc methylome. (A) Overlaps of loci whose methylation status changes differ between TN and TX relative to age (FDR < 0.05) with regions of differing CpG densities. (B) Overlaps of loci whose methylation status changes differ between TN and TX bass relative to age (FDR < 0.05) with gene regions. Asterisks (*) indicate significant enrichment or depletions in these regions relative to background sites for loci whose changes with age are lower (blue) or higher (red) for TN compared to TX bass, with bars indicating significant differences between loci.
**Figure S3:** Interactions between sex and location across the LBc methylome. (A) Overlaps of loci whose methylation status differences between male and female bass change between TN compared to TX (FDR < 0.05) with regions of differing CpG densities. (B) Overlaps of loci whose methylation status differences between male and female bass change between TN compared to TX (FDR < 0.05) with gene regions. Asterisks (*) indicate significant enrichment or depletions in these regions relative to background sites for loci whose differences between male and female bass is lower in TN compared to TX (blue) or higher (red) for TN compared to TX bass, with bars indicating significant differences between loci.
**Figure S4:** Interactions between age, sex, and location across the LBc methylome. (A) Overlaps of loci whose changes in methylation with age differ between male and female bass depending on location (TN or TX) (FDR < 0.05) with regions of differing CpG densities. (B) Overlaps of loci whose changes in methylation with age differ between male and female bass depending on location (TN or TX) (FDR < 0.05) with gene regions. Asterisks (*) indicate significant enrichment or depletions in these regions relative to background sites for loci whose differences between male and female bass with age is lower in TN compared to TX (blue) or higher (red) for TN compared to TX bass, with bars indicating significant differences between loci.

**Data S3:** LBc annotated age DMCs. Annotations of CpG loci whose methylation status differs significantly with age. Loci with negative model coefficients indicate methylation decreases with age, and loci with positive model coefficients indicate methylation increases with age. Overlaps with differing CpG dense regions (island, shores, shelves, and open seas) and regions of differing gene contexts (promoters, exons, introns, and intergenic regions) are indicated by column names. Gene IDs, symbols, and descriptions are also provided in corresponding columns for loci overlapping with genes.

**Data S4:** LBc annotated sex DMCs. Annotations of CpG loci whose methylation status differs significantly between male and female bass. Loci with negative model coefficients indicate lower methylation in male compared to female bass, and loci with positive model coefficients indicate higher methylation in male bass compared to females. Overlaps with differing CpG dense regions (island, shores, shelves, and open seas) and regions of differing gene contexts (promoters, exons, introns, and intergenic regions) are indicated by column names. Gene IDs, symbols, and descriptions are also provided in corresponding columns for loci overlapping with genes.

**Data S5:** LBc annotated location DMCs. Annotations of CpG loci whose methylation status differs significantly between bass from Texas and Tennessee. Loci with negative model coefficients indicate lower methylation in Tennessee compared to bass from Texas, and loci with positive model coefficients indicate higher methylation in Tennessee bass compared to those from Texas. Overlaps with differing CpG dense regions (island, shores, shelves, and open seas) and regions of differing gene contexts (promoters, exons, introns, and intergenic regions) are indicated by column names. Gene IDs, symbols, and descriptions are also provided in corresponding columns for loci overlapping with genes.

**Data S6:** LBc annotated age sex DMCs. Annotations of CpG loci whose methylation status with age differs significantly between male and female bass belonging to the LBc. Loci with negative model coefficients indicate that changes in methylation with age are lower for male compared to female largemouth bass. Loci with positive model coefficients indicate methylation changes with age are higher for male compared to female bass. Overlaps with differing CpG dense regions (island, shores, shelves, and open seas) and regions of differing gene contexts (promoters, exons, introns, and intergenic regions) are indicated by column names. Gene IDs, symbols, and descriptions are also provided in corresponding columns for loci overlapping with genes.

**Data S7:** LBc annotated age location DMCs. Annotations of CpG loci whose methylation status with age differs significantly between Texas and Tennessee bass. Loci with negative model coefficients indicate that changes in methylation with age are lower for Tennessee compared to Texas bass. Loci with positive model coefficients indicate methylation changes with age are higher for Tennessee compared to Texas bass. Overlaps with differing CpG dense regions (island, shores, shelves, and open seas) and regions of differing gene contexts (promoters, exons, introns, and intergenic regions) are indicated by column names. Gene IDs, symbols, and descriptions are also provided in corresponding columns for loci overlapping with genes.

**Data S8:** LBc annotated sex Location DMCs. Annotations of CpG loci whose differences in methylation status between male and female largemouth bass change with location, specifically between Texas and Tennessee populations. Loci with negative model coefficients indicate that differences in methylation between male and female bass are lower for Tennessee compared to Texas populations. Loci with positive model coefficients indicate that differences in methylation between male and female bass are greater for Tennessee compared to Texas populations. Overlaps with differing CpG dense regions (island, shores, shelves, and open seas) and regions of differing gene contexts (promoters, exons, introns, and intergenic regions) are indicated by column names. Gene IDs, symbols, and descriptions are also provided in corresponding columns for loci overlapping with genes.

**Data S9:** LBc annotated age sex location DMCs. Annotations of CpG loci whose differences in methylation with age between male and female bass is influenced by location. Loci with negative model coefficients indicate that as methylation changes with age in male and female bass, differences in slopes of these changes are lower for Tennessee compared to Texas populations. Positive model coefficients indicate slopes are greater for Tennessee bass compared to those from Texas. Overlaps with differing CpG dense regions (island, shores, shelves, and open seas) and regions of differing gene contexts (promoters, exons, introns, and intergenic regions) are indicated by column names. Gene IDs, symbols, and descriptions are also provided in corresponding columns for loci overlapping with genes.

**Data S10:** LBc CpG clock. Annotated epigenetic model predicting age across the LBc using elastic net regression. This model was trained on 124 individuals (~80% of the total dataset) and tested on 32 naïve samples (~20% of the total dataset) using CpG loci.

**Data S11:** LBc regional clock. Annotated epigenetic model predicting age across the LBc using elastic net regression on 200 bp regions of the genome that gain or lose methylation with age. This model was trained on 124 individuals (~80% of the total dataset) and tested on 32 naïve samples (~20% of the total dataset).

**Data S12:** LBc model errors. CSV file containing all chronological and predicted ages with resulting errors from each model (CpG and 200 bp regions) used to estimate age across the LBc. Locations and sex of each individual used in test data, mean and median absolute errors, and correlations of predicted ages with chronological ages for bass are also included.

## Data Availability

All data needed to evaluate conclusions of this manuscript are present in the paper and/or the Supporting Information. Raw read files, along with the processed methylation matrix generated by the enzymatic methylation sequencing, are available via GEO accession GSE343518 and Department of Energy Office of Science and Technical Information, doi: 10.13139/ORNLNCCS/3818139.
