# Supplementary material for: Epigenetic Clocks Can Predict Age in the Largemouth Bass‐Complex Despite Spatial and Sexual Variation
Source: Evol Appl. 2026 Oct 14;19(10):e70328. doi: 10.1111/eva.70328 (PMC13668047; doi:10.1111/eva.70328)
Supplement: Supplementary file 1 [file EVA-19-e70328-s003.docx]

**Supplemental Information for:**

**Universal epigenetic clocks can predict age in the Largemouth Bass-complex despite spatial and sexual variation**

Steven T. Gardner, Daniel J. Daugherty, Dijar Lutz-Carrillo, Brenda Pracheil, and Kristine N. Moody

*Corresponding author. Email: gardnerst@ornl.gov

| **Supplemental Figure 1** | Page 2 |
| --- | --- |
| **Supplemental Figure 2** | Page 3 |
| **Supplemental Figure 3** | Page 4 |
| **Supplemental Figure 4** | Page 5 |
| **Supplemental File 1** | Page 6 |
| **Supplemental File 2** | Page 7 |
| **Supplemental File 3** | Page 8 |
| **Supplemental File 4** | Page 8 |
| **Supplemental File 5** | Page 8 |
| **Supplemental File 6** | Page 8 |
| **Supplemental File 7** | Page 9 |
| **Supplemental File 8** | Page 9 |
| **Supplemental File 9** | Page 9 |
| **Supplemental File 10** | Page 9 |
| **Supplemental File 11** | Page 10 |
| **Supplemental File 12** | Page 10 |

**Table of Contents:**

Fig. S1. Interactions between age and sex across the LBc methylome

A. Overlaps of loci whose methylation status changes differ between males and females relative to age (FDR < 0.05) with regions of differing CpG densities. B. Overlaps of loci whose methylation status changes differ between males and females relative to age (FDR < 0.05) with gene regions. Asterisks (*) indicate significant enrichment or depletions of loci in these regions relative to background sites for loci whose changes are lower (blue) or higher for males (red) compared to females, with bars indicating significant differences between loci. C. Enriched pathways for loci whose methylation changes are lower for males (blue) with age compared to females.

**Fig. S2. Interactions between age and location across the LBc methylome**

A. Overlaps of loci whose methylation status changes differ between TN and TX relative to age (FDR < 0.05) with regions of differing CpG densities. B. Overlaps of loci whose methylation status changes differ between TN and TX bass relative to age (FDR < 0.05) with gene regions. Asterisks (*) indicate significant enrichment or depletions in these regions relative to background sites for loci whose changes with age are lower (blue) or higher (red) for TN compared to TX bass, with bars indicating significant differences between loci.

Fig. S3. Interactions between sex and location across the LBc methylome

A. Overlaps of loci whose methylation status differences between male and female bass change between TN compared to TX (FDR < 0.05) with regions of differing CpG densities. B. Overlaps of loci whose methylation status differences between male and female bass change between TN compared to TX (FDR < 0.05) with gene regions. Asterisks (*) indicate significant enrichment or depletions in these regions relative to background sites for loci whose differences between male and female bass is lower in TN compared to TX (blue) or higher (red) for TN compared to TX bass, with bars indicating significant differences between loci.

**Fig. S4. Interactions between age, sex, and location across the LBc methylome**

A. Overlaps of loci whose changes in methylation with age differ between male and female bass depending on location (TN or TX) (FDR < 0.05) with regions of differing CpG densities. B Overlaps of loci whose changes in methylation with age differ between male and female bass depending on location (TN or TX) (FDR < 0.05) with gene regions. Asterisks (*) indicate significant enrichment or depletions in these regions relative to background sites for loci whose differences between male and female bass with age is lower in TN compared to TX (blue) or higher (red) for TN compared to TX bass, with bars indicating significant differences between loci.

**Data S1.** **LBc Metadata (separate file)**

Metadata listing otolith ages, location of origin, sex, specific capture location, and GPS coordinates of bass belonging to the LBc used to assess factors influencing the methylome variation and construct nonlethal epigenetic clocks.

Data S2. Overlaps of differentially methylated CpG loci with differing regions of CpG density and gene regions according to age, sex, and location. Background loci are in bold, with symbols: (-) and (+) indicating loss or gain in methylation.

|  | Regions of CpG Density | | | | | | | |
| --- | --- | --- | --- | --- | --- | --- | --- | --- |
|  | Islands | | Shores | | Shelves | | Open Seas | |
| Background # | **36941** | | **80364** | | **68950** | | **1792089** | |
| Model term | (-) | (+) | (-) | (+) | (-) | (+) | (-) | (+) |
| Age | 9 | 16 | 35 | 33 | 25 | 20 | 752 | 424 |
| Sex | 2 | 0 | 4 | 0 | 3 | 0 | 47 | 8 |
| Location | 306 | 927 | 456 | 1998 | 403 | 1567 | 9244 | 40364 |
| Age*Sex | 94 | 5 | 151 | 13 | 106 | 11 | 2291 | 297 |
| Age*  Location | 3 | 0 | 14 | 2 | 6 | 1 | 189 | 30 |
| Sex*  Location | 2 | 0 | 4 | 0 | 3 | 0 | 47 | 8 |
| Age*Sex*  Location | 0 | 0 | 0 | 0 | 0 | 0 | 8 | 10 |
|  | **Gene Regions** | | | | | | | |
|  | Introns | | Exons | | Promoters | | Intergenic | |
| Background # | **1054315** | | **258828** | | **98547** | | **566654** | |
| Model term | (-) | (+) | (-) | (+) | (-) | (+) | (-) | (+) |
| Age | 368 | 224 | 89 | 79 | 35 | 55 | 329 | 135 |
| Sex | 22 | 4 | 8 | 2 | 4 | 0 | 22 | 2 |
| Location | 4990 | 22822 | 872 | 3742 | 696 | 2441 | 3851 | 15851 |
| Age*Sex | 1444 | 157 | 380 | 31 | 119 | 21 | 699 | 117 |
| Age*  Location | 111 | 19 | 28 | 3 | 16 | 0 | 57 | 11 |
| Sex*  Location | 22 | 4 | 8 | 2 | 4 | 0 | 22 | 2 |
| Age*Sex*  Location | 4 | 7 | 1 | 1 | 1 | 0 | 2 | 2 |

**Data S3.** **LBc Annotated Age DMCs (separate file)**

Annotations of CpG loci whose methylation status differs significantly with age. Loci with negative model coefficients indicate methylation decreases with age, and loci with positive model coefficients indicate methylation increases with age. Overlaps with differing CpG dense regions (island, shores, shelves, and open seas) and regions of differing gene contexts (promoters, exons, introns, and intergenic regions) are indicated by column names. Gene IDs, symbols, and descriptions are also provided in corresponding columns for loci overlapping with genes.

**Data S4. LBc Annotated Sex DMCs (separate file)**

Annotations of CpG loci whose methylation status differs significantly between male and female bass. Loci with negative model coefficients indicate lower methylation in male compared to female bass, and loci with positive model coefficients indicate higher methylation in male bass compared to females. Overlaps with differing CpG dense regions (island, shores, shelves, and open seas) and regions of differing gene contexts (promoters, exons, introns, and intergenic regions) are indicated by column names. Gene IDs, symbols, and descriptions are also provided in corresponding columns for loci overlapping with genes.

**Data S5. LBc Annotated Location DMCs (separate file)**

Annotations of CpG loci whose methylation status differs significantly between bass from Texas and Tennessee. Loci with negative model coefficients indicate lower methylation in Tennessee compared to bass from Texas, and loci with positive model coefficients indicate higher methylation in Tennessee bass compared to those from Texas. Overlaps with differing CpG dense regions (island, shores, shelves, and open seas) and regions of differing gene contexts (promoters, exons, introns, and intergenic regions) are indicated by column names. Gene IDs, symbols, and descriptions are also provided in corresponding columns for loci overlapping with genes.

**Data S6. LBc Annotated Age Sex DMCs (separate file)**

Annotations of CpG loci whose methylation status with age differs significantly between male and female bass belonging to the LBc. Loci with negative model coefficients indicate that changes in methylation with age are lower for male compared to female largemouth bass. Loci with positive model coefficients indicate methylation changes with age are higher for male compared to female bass. Overlaps with differing CpG dense regions (island, shores, shelves, and open seas) and regions of differing gene contexts (promoters, exons, introns, and intergenic regions) are indicated by column names. Gene IDs, symbols, and descriptions are also provided in corresponding columns for loci overlapping with genes.

**Data S7. LBc Annotated Age Location DMCs (separate file)**

Annotations of CpG loci whose methylation status with age differs significantly between Texas and Tennessee bass. Loci with negative model coefficients indicate that changes in methylation with age are lower for Tennessee compared to Texas bass. Loci with positive model coefficients indicate methylation changes with age are higher for Tennessee compared to Texas bass. Overlaps with differing CpG dense regions (island, shores, shelves, and open seas) and regions of differing gene contexts (promoters, exons, introns, and intergenic regions) are indicated by column names. Gene IDs, symbols, and descriptions are also provided in corresponding columns for loci overlapping with genes.

Data S8. LBc Annotated Sex Location DMCs (separate file)

Annotations of CpG loci whose differences in methylation status between male and female largemouth bass change with location, specifically between Texas and Tennessee populations. Loci with negative model coefficients indicate that differences in methylation between male and female bass are lower for Tennessee compared to Texas populations. Loci with positive model coefficients indicate that differences in methylation between male and female bass are greater for Tennessee compared to Texas populations. Overlaps with differing CpG dense regions (island, shores, shelves, and open seas) and regions of differing gene contexts (promoters, exons, introns, and intergenic regions) are indicated by column names. Gene IDs, symbols, and descriptions are also provided in corresponding columns for loci overlapping with genes.

Data S9. LBc Annotated Age Sex Location DMCs (separate file)

Annotations of CpG loci whose differences in methylation with age between male and female bass is influenced by location. Loci with negative model coefficients indicate that as methylation changes with age in male and female bass, differences in slopes of these changes are lower for Tennessee compared to Texas populations. Positive model coefficients indicate slopes are greater for Tennessee bass compared to those from Texas. Overlaps with differing CpG dense regions (island, shores, shelves, and open seas) and regions of differing gene contexts (promoters, exons, introns, and intergenic regions) are indicated by column names. Gene IDs, symbols, and descriptions are also provided in corresponding columns for loci overlapping with genes.

**Data S10. LBc CpG Clock (separate file)**

Annotated epigenetic model predicting age across the LBc using elastic net regression. This model was trained on 124 individuals (~80% of the total dataset) and tested on 32 naïve samples (~20% of the total dataset) using CpG loci.

**Data S11. LBc Regional Clock (separate file)**

Annotated epigenetic model predicting age across the LBc using elastic net regression on 200 bp regions of the genome that gain or lose methylation with age. This model was trained on 124 individuals (~80% of the total dataset) and tested on 32 naïve samples (~20% of the total dataset).

Data S12. LBc Mode;l Errors (separate file)

CSV file containing all chronological and predicted ages with resulting errors from each model (CpG and 200bp regions) used to estimate age across the LBc. Locations and sex of each individual used in test data, mean and median absolute errors, and correlations of predicted ages with chronological ages for bass are also included.
